# Hughes-Stovin-Syndrom: eine lebensbedrohliche Manifestation des Behçet-Syndroms

**DOI:** 10.1007/s00393-023-01371-0

**Published:** 2023-06-06

**Authors:** Nikolas Ruffer, Martin Krusche, Konstanze Holl-Ulrich, Fabian Lötscher, Ina Kötter

**Affiliations:** 1 III. Medizinische Klinik und Poliklinik, Universitätsklinikum Hamburg-Eppendorf, Martinistr. 52, 20246 Hamburg, Deutschland; 2 Konsultations- und Referenzzentrum für Vaskulitis-Diagnostik, Labor Lademannbogen MVZ GmbH, Hamburg, Deutschland; 3 Universitätsklinik für Rheumatologie und Immunologie, Inselspital, Universitätsspital Bern, Bern, Schweiz; 4 Klinik für Rheumatologie und Immunologie, Klinikum Bad Bramstedt, Bad Bramstedt, Deutschland

**Keywords:** Vaskulitis, Pulmonalarterienaneurysma, Pulmonalarterienthrombose, Tiefe Venenthrombose, Hämoptysen, Vasculitis, Pulmonary artery aneurysm, Pulmonary artery thrombosis, Deep vein thrombosis, Hemoptysis

## Abstract

Das Hughes-Stovin-Syndrom (HSS) ist eine entzündliche Systemerkrankung unklarer Genese, die inzwischen dem Spektrum des Behçet-Syndroms (BS) zugeordnet wird. Wegweisende Befunde sind rezidivierende Thrombosen des venösen Systems und oberflächliche Thrombophlebitiden in Kombination mit beidseitigen Pulmonalarterienaneurysmen (PAA). Die Pulmonalisangiographie mittels Computertomographie ist von entscheidender diagnostischer Bedeutung, um die (entzündliche) Beteiligung der Pulmonalarterien darzustellen. Die Therapie des HSS orientiert sich an den Empfehlungen der European Alliance of Associations for Rheumatology (EULAR) für das BS und sieht primär eine Immunsuppression mit Cyclophosphamid und Glukokortikoiden vor. Neben einer medikamentösen Therapie sollte eine interventionelle Versorgung der PAA evaluiert werden. Eine spontane PAA-Ruptur muss auch bei Remission der Erkrankung und/oder deutlicher Regredienz des PAA-Durchmessers aufgrund einer fragilen Gefäßarchitektur bedacht werden.

## Abstract (en)

Hughes-Stovin syndrome (HSS) is a systemic inflammatory condition of unknown origin that is considered to be part of the Behçet’s syndrome (BS) spectrum. Recurrent venous thrombosis and superficial thrombophlebitis in combination with bilateral pulmonary artery aneurysms (PAA) represent the hallmark of HSS. The diagnostic evaluation includes computed tomography pulmonary angiography to detect signs of pulmonary vasculitis. The management of HSS is based on the European Alliance of Associations for Rheumatology (EULAR) recommendations for BS and mainly comprises immunosuppressive therapy with glucocorticoids and cyclophosphamide. In addition to drug therapy, PAA should be evaluated for interventional treatment. Spontaneous PAA rupture due to fragile vessel architecture can occur even in cases of remission and/or PAA regression.

Im Jahr 1959 erkannten Hughes und Stovin [1] erstmals die seltene Assoziation von venösen Thrombosen und Pulmonalarterienaneurysmen (PAA). Die Autoren berichten von 4 jungen Patienten^1^ (14 bis 35 Jahre) mit tiefen Venenthrombosen (TVT) und rezidivierendem Fieber, die letztendlich an der Ruptur eines PAA mit Bronchusperforation verstarben. Interessanterweise entwickelte Patient 1 trotz Antikoagulation weitere Venenthrombosen. Bei der Sektion fanden sich thrombotische Veränderungen mit partieller Rekanalisierung im Bereich der PAA. Die histologische Untersuchung zeigte eine ausgeprägte Destruktion elastischer und muskulärer Fasern. In der Media präsentierten sich entzündliche Infiltrate, die bis in den Thrombus und die Adventitia reichten.

Die seltene Kombination aus Beteiligung des venösen und arteriellen Systems im Kontext einer systemischen Entzündung ist ein spezifisches Kennzeichen des Behçet-Syndroms (BS) [4]. Während eine pulmonale Beteiligung grundsätzlich selten beim BS auftritt, sind die Pulmonalarterien hierbei wiederum häufig betroffen [5, 6]. Neben der Bildung von PAA können sich dabei auch (ortsständige) Pulmonalarterienthrombosen (PAT) und eine pulmonale Vaskulitis (PV) entwickeln [6]. Interessanterweise treten die PAA überwiegend bei jungen Männern während der Frühphase der Erkrankung auf und sind stark mit TVT assoziiert [7, 8].

## Hughes-Stovin-Syndrom

**Figure 1:**
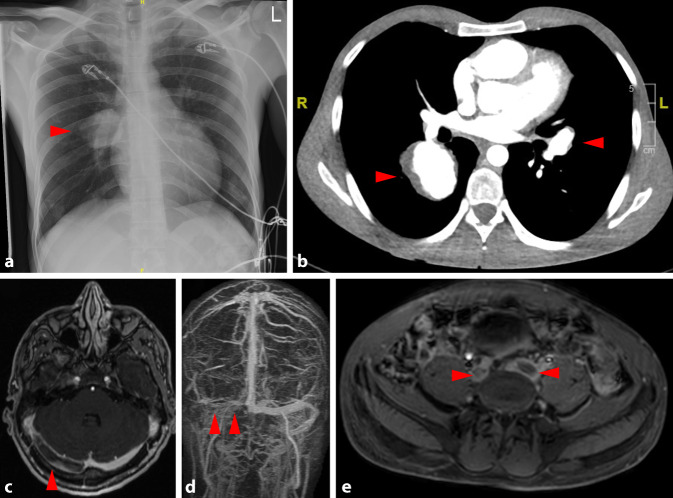

Das Hughes-Stovin-Syndrom^2^ (HSS) ist eine entzündliche Systemerkrankung unklarer Genese, die inzwischen dem Spektrum des BS zugeordnet wird [4, 10, 11]. Da typische Zeichen des BS (z. B. orogenitale Aphthen, Uveitis) häufig fehlen, wird auch die Bezeichnung „inkomplettes BS“ verwendet [11]. Wegweisende Befunde sind rezidivierende Thrombosen des venösen Systems und oberflächliche Thrombophlebitiden in Kombination mit beidseitigen PAA (Abb. 1; [8, 12]). Aus pathophysiologischer Sicht handelt es sich um eine systemische Vaskulitis, die vorwiegend periphere Venen und die Pulmonalarterien (pulmonale Vaskulitis) befällt [12]. Weniger als 100 Fälle sind bisher in der medizinischen Literatur veröffentlicht worden [13].

### Klinik

Das HSS betrifft überwiegend junge Männer (Geschlechterverhältnis etwa 3:1) [8]. Anamnestisch schildern die Betroffenen vorwiegend Husten bzw. Hämoptysen, Dyspnoe, rezidivierendes Fieber und Gewichtsverlust. Orale (19,3 %) oder genitale Ulzera (10,5 %) finden sich im Gegensatz zum klassischen BS selten [8]. Eine okuläre Inflammation ist ungewöhnlich. Emad et al. [8] fanden in ihrer systematischen Übersichtsarbeit keinen Betroffenen mit einer Uveitis.

Die Affektion der Pulmonalarterien tritt fast ausschließlich beidseitig auf, wobei die Lappenarterien am häufigsten betroffen sind und die Hauptstämme der PA nur in etwa einem Drittel der Fälle [8]. Etwa 85 % der Betroffenen entwickeln TVT, die sich typischerweise im Bereich der *V. femoralis communis*, der *V. poplitea* oder *V. cava inferior* manifestiert [8]. Zusätzlich können sich auch Thrombophlebitiden und Sinusvenenthrombosen entwickeln [8].

In der Übersichtsarbeit von Emad et al. [8] verstarb etwa ein Fünftel der Betroffenen durch massive Hämoptysen, die in den meisten Fällen von einem rupturierten Pseudoaneurysma der PA (PAP) ausgingen.

#### Klassifikationskriterien.

Da bisher keine diagnostischen Kriterien für das HSS^3^ etabliert wurden, hat die HSS International Study Group (HSSISG) im Rahmen einer systematischen Literaturrecherche Klassifikationskriterien entwickelt (Tab. 1). Hierfür werden (1) thrombotische Ereignisse, (2) ein normales Gerinnungsprofil und (3) Zeichen einer pulmonalen Vaskulitis anhand einer Pulmonalisangiographie mittels Computertomographie (CTPA) gefordert.

**Table 1:** 

(a)	Thrombotische Manifestationen des venösen oder arteriellen Systems: rezidivierende Thrombophlebitiden, tiefe Venenthrombosen, Sinusvenenthrombosen, intrakardiale Thromben, arterielle Thrombosen
(b)	Normales Gerinnungsprofil: Anti-Cardiolipin-Antikörper, β2-Glykoprotein, Faktor-V-Leiden, Prothrombin, Protein C und S
(c)	Computertomographie der Pulmonalarterien/Pulmonalisangiographie (CTPA): Zeichen pulmonalarterieller Aneurysmen (mit oder ohne intraaneurysmaler In-situ-Thrombose), Kontrastmittelanreicherung im Bereich der pulmonalarteriellen Gefäßwand

### Diagnostik

Die Diagnose des HSS beruht in erster Linie auf dem klinischen Bild einer entzündlichen Systemerkrankung mit gleichzeitiger Affektion des venösen und arteriellen Systems, die sich durch rezidivierende Thrombosen und Zeichen einer pulmonalen Vaskulitis manifestiert. Die Klassifikationskriterien [8] und der Referenzatlas^4^ [12] der HSSISG können den diagnostischen Prozess unterstützen. Die diagnostischen Prinzipien bei der Abklärung eines BS^5^ können ebenfalls hilfreich sein [15, 16]. Ferner sollten konkurrierende Ursachen (insbesondere Gerinnungsstörungen) ausgeschlossen werden.

Die Pulmonalisangiographie mittels Computertomographie (CTPA) ist von entscheidender Bedeutung, um die entzündliche Beteiligung der PA darzustellen (Tab. 2; [12]): Typische Befunde sind in diesem Zusammenhang eine Kontrastmittelanreicherung im Bereich der PA-Gefäßwand (Frühzeichen einer pulmonalen Vaskulitis), aneurysmatische Veränderungen der PA und Bronchialarterien, intraaneurysmale In-situ-Thrombosen (PAT), Pseudoaneurysmen der PA (PAP) sowie Rechtsherzbelastungszeichen.

**Table 2:** 

I	Kontrastmittelanreicherung im Bereich der Gefäßwand des Pulmonalarterienaneurysmas (PAA)
II	Echtes „stabiles“ PAA
III	Instabiles PAA
IV	Pulmonalarterielles Pseudoaneurysma (PAP)
V	Instabiles PAP
VI	Rechtsherzbelastungszeichen mit oder ohne intrakardialer Thrombose

Differenzialdiagnostisch bedeutsam ist die Unterscheidung zwischen ortsständiger (In-situ‑)Thrombosierung im Bereich der PAA, die pathophysiologisch von einer Lungenarterienembolie abgrenzt werden sollte, insbesondere bei gleichzeitigem Nachweis von TVT [12, 17, 18].

Während bei vermutetem HSS eine CT mit Pulmonalisangiographie zwingend indiziert ist, gehört diese Untersuchung nicht grundsätzlich zur Routinediagnostik beim BS. Beim BS genügt daher zunächst eine Röntgenuntersuchung des Thorax, die größere PAA adressiert [19]. Allerdings sollte bei entsprechenden Symptomen wie Husten, Dyspnoe, Hämoptysen, B‑Symptomatik oder auch unerklärten erhöhten Entzündungsparametern an die Möglichkeit von PAA gedacht und die entsprechende Bildgebung veranlasst werden.

Spezifische Laborveränderungen lassen sich beim HSS nicht nachweisen. Laboranalytisch finden sich häufig deutlich erhöhte serologische Entzündungsparameter und eine Anämie. Antinukleäre Antikörper (ANA), antineutrophile zytoplasmatische Antikörpern (ANCA), Antiphospholipidantikörper und ggf. IgG-Subklassen^6^ sollten aus differenzialdiagnostischen Erwägungen bestimmt werden. Eine Luesserologie (Differenzialdiagnostik), ein Interferon-Gamma-Test (potenzielle Therapie mit TNF-α-Antagonisten) sowie ein HIV-, Hepatitis-B- und -C-Screening (immunsuppressive Therapie) sollten ergänzt werden.

### Histopathologie

Bereits Hughes und Stovin [1] schilderten entzündliche Veränderungen im Bereich der aneurysmatischen PA-Wand (Infiltration durch Schaumzellen, Plasmazellen und Lymphozyten), die sich bis in den angrenzenden Thrombus des PAA erstreckten. Pirani et al. [3] beschrieben ebenfalls ein lymphozytäres Infiltrat im Bereich der PAA-Wand.

Angesichts der Seltenheit dieses BS-Phänotyps liegen bisher nur vereinzelte Berichte zur Histologie der PAA vor. Für rupturierte PAA bei BS sind eine Thrombusbildung mit partieller Rekanalisierung und perivaskuläre Infiltrate bestehend aus mononukleären Zellen beschrieben worden [21]. Spezifische histopathologische Merkmale sind für das BS mit Gefäßbeteiligung bisher jedoch nicht berichtet worden [22].

**Figure 2:**
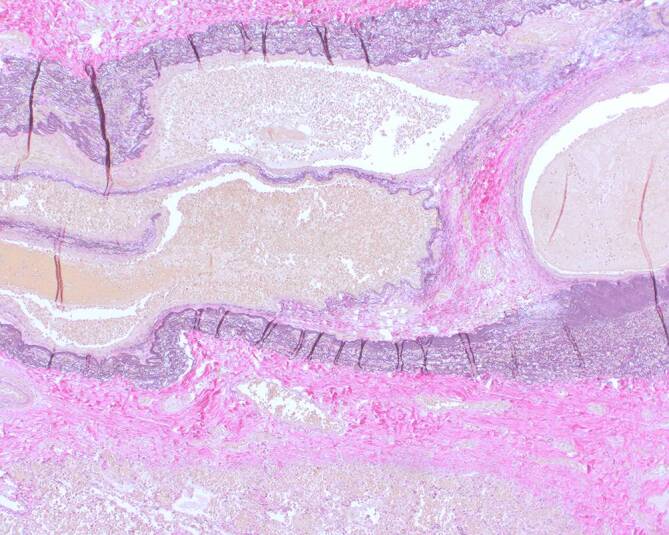

Im Gegensatz zu „echten“ Aneurysmata, die aus einer sackförmigen Erweiterung aller Gefäßwandschichten bestehen, finden sich jedoch beim HSS häufig sog. „Pseudoaneurysmen“ (Aneurysma spurium), die durch entzündliche Destruktion zu einer Aufsplitterung der Gefäßwand mit „falschen“ blutführenden Lichtungen in tieferen Schichten wie Media und Adventitia führen (Abb. 2). Diese dünne entzündlich strukturgeschädigte Wand derartiger PAP führt daher zu einem deutlich höheren Rupturrisiko [8].

### Therapie

Die Therapie des HSS orientiert sich im Wesentlichen an den Empfehlungen der European Alliance of Associations for Rheumatology (EULAR) für das BS [23]. Für die Therapie vaskulärer Läsionen kommen in erster Linie Immunsuppressiva zum Einsatz (Empfehlungen 4 bis 6), da diese Läsionen als Ausdruck einer entzündlichen (vaskulitischen) Aktivität gelten. Das Vorliegen von PAA bedingt in diesem Zusammenhang die Indikation zur Therapie mit hoch dosierten Glukokortikoiden und Cyclophosphamid (CYC). In refraktären Fällen kann auch der Einsatz von TNF-α-Antagonisten (Infliximab) erwogen werden [8, 23–26]. Sogar eine deutliche Regredienz oder Normalisierung der Gefäßarchitektur ist für beide Therapieansätze (CYC, TNF-α-Antagonisten) beschrieben worden [22, 26, 27].

Wesentliche therapeutische Konflikte ergeben sich durch teilweise schwerwiegende thrombotische Ereignisse (insbesondere Thrombose der V. cava inferior) bei gleichzeitig hohem Blutungsrisiko durch die PAA (ergänzende Antikoagulation?). Zusätzlich kann die Indikationsstellung zur interventionellen Versorgung im Kontext einer hochgradig aktiven Erkrankung mit großer Unsicherheit behaftet sein. In jedem Fall sollte eine interdisziplinäre Behandlung an einem rheumatologischen Zentrum mit Zugang zu herz- und gefäßchirurgischer Expertise erfolgen.

#### Antikoagulation.

Für das BS (und HSS) sind der therapeutische Nutzen und das Sicherheitsprofil einer Antikoagulation nicht abschließend untersucht und daher Gegenstand einer kontroversen Diskussion [28]. Im Allgemeinen gelten (rezidivierende) thrombotische Ereignisse als Ausdruck der entzündlichen Krankheitsaktivität, die primär durch eine systemische Immunsuppression behandelt werden sollte. Basierend auf retrospektiven Studien scheint eine ergänzende Antikoagulation zur Rezidivprophylaxe beim BS keinen Zusatznutzen zu bewirken [23]. Systematische Studien zu dieser Fragestellung liegen jedoch nicht vor und sind dringend notwendig [22]. Allerdings könnte die Auftretenshäufigkeit von postthrombotischen Syndromen unter Antikoagulation reduziert sein [23]. Hierbei betonen die EULAR-Empfehlungen das Risiko von Blutungen unter Antikoagulation bei gleichzeitigem Vorliegen von PAA, die jedoch im Falle einer Therapie ausgeschlossen sein sollten [23]. Auf der anderen Seite erhielt die Mehrheit der HSS-Patienten (59,6 %) in der Arbeit von Emad et al. [8] eine Antikoagulation. In Fällen mit schwerwiegenden und progredienten Thrombosen des venösen Systems unter bereits bestehender Immunsuppression ergibt sich jedoch ein klinisches Dilemma, das letztendlich eine äußerst herausfordernde Einzelfallentscheidung darstellt und im interdisziplinären Team diskutiert werden sollte [29].

#### Chirurgische und endovaskuläre Verfahren.

Zur Therapie von PAA wird primär der Einsatz von Cyclophosphamid empfohlen, und der Einsatz chirurgischer Verfahren (z. B. Lobektomie) ist nur im Fall von lebensbedrohlichen Situationen vorgesehen [22, 23]. Bei hohem Blutungsrisiko können – bei geeigneter Größe der Läsion – auch endovaskuläre Verfahren (Embolisation oder Stenting) eingesetzt werden [23, 30]. Hämoptysen können sekundär im Rahmen einer Bronchialarterienhyperplasie auftreten, die eine Komplikation der PA-Beteiligung darstellt und als Blutungsquelle bedacht werden sollte [30, 31]. Grundsätzlich sollte eine medikamentöse Therapie der Versorgung von Aneurysmen vorausgehen, sodass eine Versorgung idealerweise im Zustand einer Remission vorgenommen werden kann. Bei aktiver Erkrankung kann eine chirurgische Versorgung durch Aneurysmen im Bereich der Anastomosen, Ruptur weiterer PAA und arteriovenöse Fisteln erheblich verkompliziert werden [30, 32–34]. Synthetische Materialien sollten bevorzugt werden, da venöse Grafts ein höheres Risiko thrombotischer Komplikationen aufweisen [23]. Als Indikation zur chirurgischen Versorgung von PAA [35] gelten beispielsweise ein PAA-Durchmesser ≥ 5,5 cm, ein kurzfristiger Progress oder Zeichen der Ruptur (Tab. 3). Aus unserer eigenen Erfahrung [29] stellt sich zusätzlich – auch bei Remission und/oder Regredienz der PAA bzw. PAP – die Frage nach einer „definitiven Versorgung“, da die Gefäßwand der Aneurysmen durch die (abgelaufenen) Inflammationsprozesse unter Umständen irreparabel geschädigt ist und aufgrund einer fragilen Gefäßarchitektur zur spontanen Ruptur (Abb. 2) neigt. Strategien zur Risikoabschätzung sind hierfür bisher nicht etabliert.

**Table 3:** 

Vaskulitis variabler Gefäße
Beteiligung der Pulmonalarterien (beidseitig)
Seltener Nachweis von vaskulitischen Veränderungen bei der Untersuchung von charakteristischen Hautläsionen
Fehlen von nekrotisierenden Veränderungen
Fehlende Granulombildung
Unterschiedliche geografische Verteilung von Krankheitsausprägungen
Schwere Verläufe bei männlichen Patienten
Vorwiegende Affektion des venösen Systems
Abschwächung der Krankheitsschwere im Verlauf
Kein erhöhtes Risiko für entzündlich bedingte Atherosklerose

## Vaskuläre Manifestationen des Behçet-Syndroms

Eine vaskuläre Beteiligung^7^ findet sich bei ca. 15–50 % aller Betroffenen [5]. Das BS betrifft dabei vorwiegend das venöse System [5, 36]. In vielen Fällen entwickeln die Betroffenen Fieber oder Gewichtsverlust mit deutlich erhöhten Entzündungsparametern [22].

Als häufigste Gefäßmanifestationen gelten isolierte TVT der unteren Extremität (ca. 85 % der Betroffenen mit Gefäßmanifestationen), die v. a. bei jungen Männern auftreten und mit Thrombophlebitiden assoziiert sind [5]. Als weiteres Krankheitszeichen können Thrombosen der *V. cava* (*V. cava superior* 8,6 % bzw. *V. cava inferior* 7,8 % der Betroffenen mit Gefäßmanifestationen) und Sinusvenen (ca. 4 % der Betroffenen mit Gefäßmanifestationen) auftreten [5].

Daneben können auch arterielle Gefäße (ca. 3–5 %) affektiert sein [5, 36–38]. Am häufigsten sind dabei die PA und Aorta betroffen. Typischerweise kommt es auch zur Aneurysmabildung oder In-situ-Thrombose [5, 22, 37]. Die Arterien der Unterlappen (v. a. rechtsseitig) sollen Prädilektionsstellen darstellen [39].

## Ist das Hughes-Stovin-Syndrom eine Variante des Behçet-Syndroms?

Inwieweit das HSS eine eigenständige Entität darstellt, ist wiederholt diskutiert worden [4, 11, 38]. Vor dem Hintergrund einer nosologischen Einordnung des BS wird diese Fragstellung im Folgenden adressiert und die Auffassung, wonach das HSS eine besondere Variante (vaskulärer Phänotyp) des BS ist, dargestellt.

### Nosologische Betrachtung des Behçet-Syndroms – mehr als eine Entität?

Das BS wird in der aktuellen Chapel Hill-Nomenklatur von 2012 [40] als Vaskulitis variabler Gefäße eingeordnet, die sowohl Arterien als auch Venen betreffen kann. Im Gegensatz zu anderen Vaskulitiden stellt die vorwiegende Affektion des venösen Systems eine Besonderheit des BS dar [41]. Weitere Unterschiede (Tab. 4) sind das häufige Fehlen von vaskulitischen (nekrotisierenden) oder granulomatösen Veränderungen in Gewebeproben, das vorwiegende Auftreten vaskulärer Manifestationen bei männlichen Patienten und die geografisch^8^ verschiedenen Krankheitsausprägungen [41].

**Table 4:** 

Absoluter PAA-Durchmesser ≥ 5,5 cm
Zunahme des PAA-Durchmessers um ≥ 0,5 cm innerhalb von 6 Monaten
Kompression angrenzender Strukturen
Thrombusbildung im Aneurysmasack
Auftreten klinischer Symptome
Nachweis von Klappenpathologien oder Shuntfluss
Nachweis eines pulmonalarteriellen Hypertonus
Zeichen der Ruptur oder Dissektion

Aufgrund klinischer und immunologischer Gemeinsamkeiten wird das BS gleichzeitig auch dem Spektrum der autoinflammatorischen Erkrankungen zugeordnet [15, 43, 44]: Gründe hierfür sind u. a. der rezidivierende Verlauf, die mukokutanen Läsionen mit neutrophiler Infiltration, das Fehlen einer antigenspezifischen T‑Zell-Antwort bzw. spezifischer Autoantikörper, die Aktivierung proinflammatorischer Zytokine (Interleukin-1) und die individuelle Prädisposition durch ein genetisch determiniertes Merkmal (humanes Leukozytenantigen B*51). Letzteres hat auch zur Auffassung als „MHC-I-opathie“ beigetragen. Da jedoch auch signifikante Unterschiede zu „klassischen“ autoinflammatorischen Erkrankungen (z. B. häufig monogenetische Erkrankung, Beginn im Kindesalter, konstant progredienter Verlauf) bestehen, ist auch diese Einordnung kontrovers diskutiert worden [15, 43, 44].

Obwohl orogenitale Aphthen und Uveitis wegweisende klinische Zeichen des BS darstellen, entwickeln keineswegs alle Patienten diese klinische Trias. Vielmehr konnten durch Assoziationsstudien verschiedene Krankheitscluster [7, 36, 45] identifiziert werden, die durch unterschiedliche dominante Befunde gekennzeichnet sind (Tab. 5). Beispielsweise tritt eine parenchymatöse Beteiligung des zentralen Nervensystems („Neuro-Behçet“) hauptsächlich bei Männern auf und ist mit okulären Manifestationen verbunden [7, 36].

**Table 5:** 

Isolierte mukokutane Beteiligung
Gelenkbeteiligung (Mono‑/Oligoarthritis)
Vaskuläre Beteiligung
Okuläre Beteiligung
Parenchymatöse Beteiligung des zentralen Nervensystems
Gastrointestinale Beteiligung

Insofern könnte das BS möglicherweise mehr als eine nosologische Entität darstellen [46], sodass der deskriptive Begriff „Behçet-Syndrom“ aus den aktuellen Empfehlungen der EULAR von 2018 [23] sinnvoll erscheint.

### Hughes-Stovin-Syndrom als vaskulärer Phänotyp des Behçet-Syndroms

Die klinischen und radiologischen Überschneidungen zwischen HSS und BS sind in der medizinischen Fachliteratur kontrovers diskutiert worden und haben letztendlich Anlass gegeben, das HSS dem Spektrum des BS zuzuordnen [4]:

- gemeinsames Auftreten multipler PAA im Kontext rezidivierender thrombotischer Ereignisse (z. B. TVT, Thrombophlebitiden oder Thrombosen der PA) als spezifische Manifestation des BS^9^,
- gleichartige pulmonale Manifestationen bei HSS und BS (z. B. beidseitige Beteiligung, inflammatorisch bedingte In-situ-Thrombosen der PA mit Tendenz zur Entwicklung von aneurysmatischen Veränderungen),
- Entwicklung von atypischen („inkompletten“) Phänotypen als wesentliches Merkmal des BS (z. B. Fehlen einer okulärer Inflammation bei vaskulärem Phänotyp),
- vorwiegende Manifestation bei jungen Männern,
- histologische Gemeinsamkeiten der (thrombosierten) PAA,
- Entwicklung rezidivierender Venenthrombosen trotz Antikoagulation,
- Wirksamkeit von immunsuppressiven Therapien bei BS und HSS.

## Fazit für die Praxis

- Das Hughes-Stovin-Syndrom ist eine lebensbedrohliche Manifestation des Behçet-Syndroms (BS) und durch die Kombination aus beidseitigen Pulmonalarterienaneurysmen und tiefen Venenthrombosen gekennzeichnet.
- Die Pulmonalisangiographie mittels Computertomographie ist aktuell der Goldstandard in der Diagnostik der pulmonalen Manifestationen.
- Die Therapie des HSS orientiert sich an den Empfehlungen der European Alliance of Associations for Rheumatology für das BS (2018) und sieht primär eine Immunsuppression mit Cyclophosphamid und Glukokortikoiden vor.
- Neben einer medikamentösen Therapie sollte auch eine Evaluation zur interventionellen Versorgung der Pulmonalarterienaneurysmen (PAA) erfolgen.
- Eine spontane PAA-Ruptur muss auch bei Remission der Erkrankung und/oder deutlicher Regredienz des PAA-Durchmessers aufgrund einer fragilen Gefäßarchitektur bedacht werden.

## Footnotes

1 Hughes und Stovin schildern den Krankheitsverlauf von 2 eigenen Patienten und vergleichen diese mit Fällen von Beattie und Hall [2] sowie Pirani et al. [3].

2 Der Begriff „Hughes-Stovin-Syndrom“ geht auf Kopp und Green [9] zurück, die in ihrem Fallbericht von 1962 auf die Darstellung von Hughes und Stovin Bezug nehmen.

3 Diagnosekriterien für das Hughes-Stovin-Syndrom (HSS) werden aktuell von der HSS International Study Group erarbeitet (persönliche Mitteilung von Yasser Emad).

4 Der Referenzatlas der Hughes-Stovin syndrome International Study Group bietet eine Hilfestellung zur frühen Diagnose der pulmonalen Vaskulitis. Hierbei werden 6 verschiedene Stadien unterschieden, die anhand der korrespondierenden radiologischen Befunde illustriert werden.

5 Hierbei sollte berücksichtigt werden, dass die Diagnosekriterien des International Team for the Revision of the International Criteria for Behçet’s Disease (ITR-ICBD) von 2014 [14] unter Umständen nicht erfüllt werden.

6 In seltenen Fällen kann auch eine IgG4-assoziierte Erkrankung mit einer Beteiligung der Pulmonalarterien einhergehen [20].

7 Eine ausführliche Darstellung der pathogenetischen Prozesse findet sich bei Bettiol et al. [22].

8 In Abhängigkeit von der geografischen Region finden sich unterschiedliche klinische Ausprägungen und Prävalenzen für das Behçet-Syndrom (BS) [22, 42]: Beispielsweise scheint eine gastrointestinale Beteiligung in ostasiatischen Ländern (insbesondere Japan) deutlich häufiger aufzutreten als in der Türkei oder Mitteleuropa. Gefäßmanifestationen (TVT und PAA) finden sich wiederum in Japan deutlich seltener. Grundsätzlich tritt das BS vermehrt im Mittelmeerraum, Nahen Osten und Ostasien auf, weswegen auch der Begriff „Seidenstraßenerkrankung“ (engl. ‚Silk Road disease‘) verwendet wird.

9 Während einseitige PAA auch im Rahmen anderer Erkrankungen (z. B. Infektionen) auftreten, finden sich beidseitige PAA fast ausschließlich bei Vaskulitiden [4]. Obwohl eine vaskulitische Affektion der PA auch bei der Takayasu-Arteriitis (TA) auftreten kann, ist die Entwicklung von PAA hingegen nicht bekannt [4]. Vielmehr finden sich bei der TA stenotische oder okklusive Veränderungen [41]. Zusätzlich findet sich in der histologischen Untersuchung keine Beteiligung aller 3 Wandschichten mit Bildung von Riesenzellen und Granulomen (pathognomonisch für die TA) [41]. Für die Polyarteriitis nodosa (PAN) ist eine Lungenbeteiligung extrem untypisch und zeigt hauptsächlich unspezifische Befunde [4, 47]. Die Bildung von PAA ist nach unserem Wissen nicht verlässlich beschrieben [4] und eine entzündliche Beteiligung der PA nur in Einzelfällen berichtet [48]. De Vries et al. [49] beschreiben den Fall eines 11-jährigen Jungen, der formal die Kriterien einer juvenilen PAN erfüllte, und argumentieren, dass das HSS möglicherweise eine Variante der PAN darstelle. Einige Autor:innen [50] betrachten Lungenmanifestationen hingegen als Ausschlusskriterium für die Diagnose einer PAN. Wiederholte Überarbeitungen der Klassifikationskriterien können zu dieser Problematik beigetragen haben, da die mikroskopische Polyangiitis früher mit der PAN zusammengefasst wurde („mikroskopische Polyarteriitis“). Während das Lungenparenchym und die Niere beim BS typischerweise nicht betroffen sind, findet sich eine derartige Parenchymbeteiligung häufig bei ANCA-assoziierten Vaskulitiden, die in seltenen Fällen auch die PA betreffen können.
